# Optimal Ranges and Thresholds of Grape Berry Solar Radiation for Flavonoid Biosynthesis in Warm Climates

**DOI:** 10.3389/fpls.2020.00931

**Published:** 2020-06-23

**Authors:** Nazareth Torres, Johann Martínez-Lüscher, Etienne Porte, S. Kaan Kurtural

**Affiliations:** Department of Viticulture and Enology, University of California Davis, Davis, CA, United States

**Keywords:** anthocyanin, canopy management, kaempferol, leaf removal, methoxypyrazines, shoot thinning

## Abstract

In commercial wine grape production, canopy management practices are applied to control the source-sink balance and improve the cluster microclimate to enhance berry composition. The aim of this study was to identify the optimal ranges of berry solar radiation exposure (exposure) for upregulation of flavonoid biosynthesis and thresholds for their degradation, to evaluate how canopy management practices such as leaf removal, shoot thinning, and a combination of both affect the grapevine (*Vitis vinifera* L. cv. Cabernet Sauvignon) yield components, berry composition, and flavonoid profile. Three experiments were conducted in Oakville, CA, USA. First experiment assessed changes in the grape flavonoid content driven by four degrees of exposure. In the second experiment, individual grape berries subjected to different exposures were collected from two cultivars (Cabernet Sauvignon and Petit Verdot). The third experiment consisted of an experiment with three canopy management treatments (i) LR (removal of 5 to 6 basal leaves), (ii) ST (thinned to 24 shoots per vine), and (iii) LRST (a combination of LR and ST) and an untreated control (UNT). Berry composition, flavonoid content and profiles, and 3-isobutyl 2-methoxypyrazine were monitored during berry ripening. Although increasing canopy porosity through canopy management practices can be helpful for other purposes, this may not be the case of flavonoid compounds when a certain proportion of kaempferol was achieved. Our results revealed different sensitivities to degradation within the flavonoid groups, flavonols being the only monitored group that was upregulated by solar radiation. Within different canopy management practices, the main effects were due to the ST. Under environmental conditions given in this trial, ST and LRST hastened fruit maturity; however, a clear improvement of the flavonoid compounds (i.e., greater anthocyanin) was not observed at harvest. Methoxypyrazine berry content decreased with canopy management practices studied. Although some berry traits were improved (i.e. 2.5° Brix increase in berry total soluble solids) due to canopy management practices (ST), this resulted in a four-fold increase in labor operations cost, two-fold decrease in yield with a 10-fold increase in anthocyanin production cost per hectare that should be assessed together.

## Introduction

In vineyard production systems, canopy management practices are usually employed to control the source-sink balance and improve the cluster microclimate leading to an improved grape composition and resultant wines (Sivilotti et al., 2016). Canopy density is usually controlled during the dormant season thought the winter pruning. Additional canopy management practices may be applied during berry development. Fruit-zone leaf removal and especially, shoot thinning have been widely used in order to increase the cluster exposure to solar radiation, reduce crop load as well as decreasing the pest pressures (Terry and Kurtural, 2011; Provost and Pedneault, 2016; Sivilotti et al., 2016), increasing flavonoid content (Martínez-Lüscher et al., 2019) and diminishing herbaceous aromas (Koch et al., 2012). Nevertheless, when high air temperature and excessive radiation combine, detrimental effects on berry acidity and flavonoid content have been reported in warm climate regions (Martínez-Lüscher et al., 2017).

Leaf removal consists of removing basal leaves around the clusters in the east or north side during grape development increasing the cluster exposure to solar radiation. It is well known that an early leaf removal (before flowering) increased total soluble solids, anthocyanins, and flavonols (Tarara et al., 2008; Diago et al., 2012; Gatti et al., 2012; Pastore et al., 2013; Bogicevic et al., 2015; Cook et al., 2015; Bubola et al., 2017). However, some authors reported increases in titratable acidity in Sangiovese (Gatti et al., 2012) and Teran (Bubola et al., 2017) cultivars while other authors found decreases in acidity with basal leaf removal on Tempranillo (Diago et al., 2012). Conversely, Sivilotti et al. (2016) reported a positive effect of leaf removal applied after ﬂowering on Merlot grapevine by improving cluster integrity by reducing incidence of *Botrytis*, and lower herbaceous aromas without aﬀecting yield and cluster mass. Contrariwise, Pastore et al. (2013) reported that defoliation at veraison reduced the anthocyanin content and increased the impact of sunburn. In fact, these authors found that leaf removal induced a general delay in the transcriptional ripening program, which was particularly apparent for structural and regulatory genes involved in the anthocyanin biosynthesis.

Clearly, vineyard location, cultivar (Tardaguila et al., 2010), timing of leaf removal (Pastore et al., 2013; Sivilotti et al., 2016), method (Diago et al., 2012), and degree of leaf removal (Feng et al., 2015), the growing season (Sivilotti et al., 2016), among others, are all factors influencing how leaf removal affects grapevine berry composition and integrity.

On the other hand, shoot thinning has been related to increased cluster and berry mass and the number of berries per cluster, with a reduction on yield (Sun et al., 2012; Jogaiah et al., 2013). Conversely, Wang et al. (2019) observed that shoot thinning had relatively minor impacts on yield components because of a compensatory effect due to the lower cluster number with concomitant increase in cluster mass. Contrarily, shoot thinning practices on grapevine did not show a great impact on berry primary metabolism (Sun et al., 2012; Wang et al., 2019), however, secondary metabolites were affected by them (Terry and Kurtural, 2011). In fact, we recently reported an increase of two-fold in the flavonol content of Merlot berries when leaf or shoot removal was applied mainly by increasing the proportion of quercetin and kaempferol derivatives in detriment of the myricetin derivatives (Martínez-Lüscher et al., 2019).

Berry composition is dependent on a complex balance between compounds derived from primary and secondary metabolism. Between secondary metabolites, flavonoids (i.e., anthocyanins and flavonols) play an important role in the quality and the antioxidant properties of grapes (Torres et al., 2016; Samoticha et al., 2018) and are very responsive to environmental factors such as solar exposure (Blancquaert et al., 2019). Anthocyanin compounds are responsive of the berry color, and flavonols act as a UV shields, contribute to the wine antioxidant capacity, color stability, and hue through co-pigmentation with anthocyanins (Gómez-Míguez et al., 2006). On the other hand, the methoxypyrazines are wine key odorants contributing to their herbaceous characteristics and have been related to unripe berries and poor-quality wines when these are not part of the wine typicity (Roujou de Boubée et al., 2000). Since they can be present in grape berry and wines at high levels, they may have an important sensorial impact on wine quality (Ryona et al., 2008). Among methoxypyrazines, the 3-isobutyl-2-methoxypyrazine (IBMP) is considered the most relevant to wine flavor due to its correlation with the intensity of the bell pepper character of wines (Roujou de Boubée et al., 2000) and its content at harvest seems to be dependent of the solar exposure (Scheiner et al., 2010; Koch et al., 2012; Sivilotti et al., 2016).

The differences found in the literature about the effect of manipulating the canopy architecture on the flavonoid and aromatic content due to different solar exposure of berries in warm climates opens an important field of research. Therefore, we aimed to find the optimal ranges of berry solar exposure estimated as percent of kaempferol (Martínez-Lüscher et al., 2019) for flavonoid synthesis up regulation and the thresholds for their degradation, and to evaluate how canopy management practices such as leaf removal, shoot thinning and a combination of both affect the grapevine yield components, berry composition, flavonoid profile, and herbaceous aromas.

## Material and Methods

### Plant Material and Experimental Design

#### Experiment 1: Berry Microclimate Affect Berry Quality and Determines Berry Skin Flavonoid Composition at Harvest

**Figure 1:**
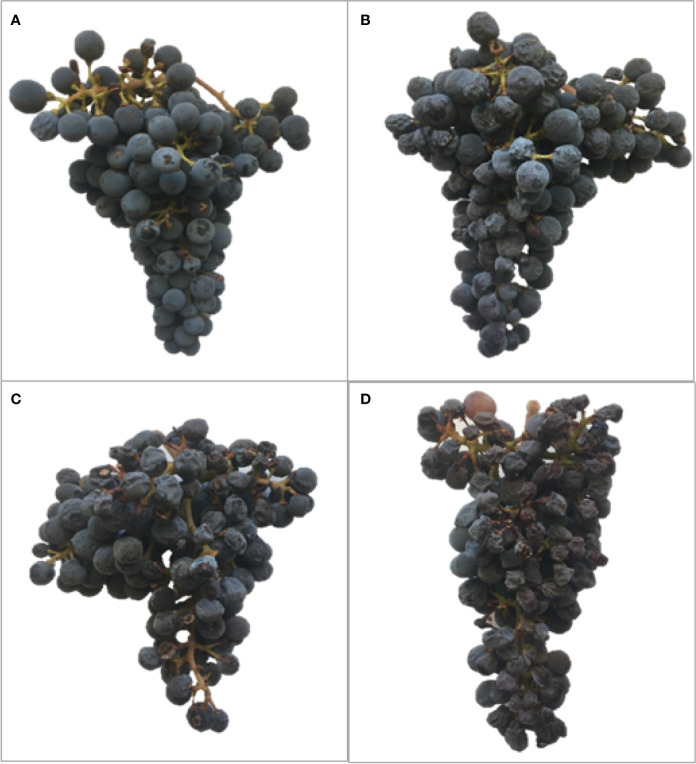
Examples of harvested clusters from Cabernet Sauvignon with different degree of exposure: **(A)** Exp− (Non exposed berries collected from clusters in canopy interior), **(B)** Exp+ Deg− (exposed but not degraded, collected from Northeast exposed clusters), **(C)** Exp+ Deg+ (exposed and degraded, collected from Southwest exposed clusters) and **(D)** Exp+ Deg++ (exposed and very degraded grapes with signs of damage collected from Southwest exposed clusters), collected in Oakville, CA in 2017.

An experiment was performed in 2017 on 7-year Cabernet Sauvignon vines (clone FPS08) grafted onto 110 Richter rootstock (*V. rupestris* x *V. berlandieri*) with NW-SE row orientation and a vine spacing of 2 m × 2.4 m (vine × row) in a commercial vineyard in Oakville, CA (38.427 N 122.410 W). Individual berries were sampled at harvest according to their position in the canopy and overexposure based on visual appearance. Each independent replicate was a sample of 75 berries collected from up to 50 plants each (200 in total), these plants being potentially the same for all exposures. From each sample, 55 berries were used for must analyses and berry mass, and the remaining 20 berries were stored at −20°C for analyses of flavonoids. Thus, four observational treatments with four replicates consisted in two rows of 25 vines each were established: (i) non-exposed berries collected from interior clusters (Exp−); (ii) exposed but free of signs of overexposure, collected from northeast exposed clusters (Exp+ Deg−); (iii) exposed and with mild signs of sunburn, collected from southwest exposed clusters (Exp+ Deg+); and (iv) exposed and with severe signs of sunburn with signs of damage collected from southwest exposed clusters (Exp+ Deg++). These treatments are provided visually in **Figure 1**.

#### Experiment 2: Relationship Between Canopy Porosity and Berry Anthocyanin and Flavonol Content in a Commercial Vineyard

In the second experiment, individual grapes from different cluster positions (interior, exposed from the west side of the canopy, exposed from the east side, and overexposed from the east side) were collected from two cultivars (Cabernet Sauvignon and Petite Verdot) grown in a commercial vineyard in Oakville, CA (38.427 N 122.410 W) in 2017. Cabernet Sauvignon grapevines (FPS clone 04 grafted onto St. George and spaced 1.2 m × 1.2 m (vine × row)) and Petit Verdot grapevines (clone 400-ENTAV-INRA grafted onto 101 to 14 Mgt and spaced 1.8 m × 1.2 m) were 21 and 9-years old, respectively. The exposure of each individual grape was estimated with fish-eye lens photography from the grape perspective pointing the zenith. The images were processed in R (version 3.2.5-6). After applying a thresholding condition to the blue channel of all images, they were converted into binary pixels (black/white). Thus, the percent of binary pixels capturing the sky was used to calculate the percentage of canopy porosity as reported previously (Martínez-Lüscher et al., 2019). Then, those berries were collected at harvest, and their flavonoid content was analyzed with reversed-phase high performance liquid chromatography. **Figure S1** shows that the % of kaempferol was a good estimator of the canopy porosity as we previously reported in Martínez-Lüscher et al. (2019).

#### Experiment 3: Response of Berry Chemistry, Flavonoid Metabolism, and Methoxypyrazine Degradation During Berry Ripening to Canopy Management Practices

The experiment was conducted in 2019 in Oakville, CA (38.428 N, 122.409 W) with row orientation NW-SE. The vineyard was spaced 2 m × 2.4 m with Cabernet Sauvignon grapevines (clone FPS08) on 110R rootstock. The grapevines were trained to a vertically shoot-positioned system with a cordon height 96 cm above vineyard floor, trained to a bilateral cordon, and pruned to 1-bud spurs. Plants were irrigated weekly with 2-drip emitters per vine, with the capacity to deliver 3.8 L of water per hour. The experiment was designed as a randomized complete block with three canopy management practices: (i) removal of 5 to 6 basal leaves on the NE side (LR); (ii) thinned to 24 shoots per vine (ST); and (iii) a combination of LR and ST (LRST) and an untreated control (no shoot thinning or leaf removal, UNT), with four replicates each consisting in 5 grapevines, 3 of which were sampled and the 2 on distal ends were treated as border plants. The ST and LR treatments were applied on 11 June 2019. Harvest commenced when the berry TSS reached to *ca*. 24°Brix on 23 September (112 DAF). The sampling time points were as follows: 2 weeks before veraison (11 July), veraison (1 August), 2 weeks after veraison (15 August), 3 weeks after veraison (20 August), 5 weeks after veraison (3 September), and harvest (23 September), were chosen to cover the response of the berry metabolism to cultural practices and the concomitant increase in exposure.

### Weather Conditions

Weather data (**Table 1**) were obtained from the California Irrigation Management Information System, CIMIS, station (#77, Oakville, CA) located on site during the growing seasons covered by the experiments and the reference period 1999 to 2019 (California Department of Water Resources, 2020). Number of days with temperatures above 30°C were counted for the 2017 and 2019 growing seasons.

**Table 1:** Weather conditions during the growing seasons of 2017, 2019 and the average for the same period in the last 20 years (1999–2019).

Month								
	April	May	June	July	August	September	October	
*Year*	Mean daily temperature (°C)							Mean
2017	20.6	22.9	18.6	17.8	22.2	29.6	18.8	21.5
2019	12.9	15.3	17.3	17.2	15.4	22.6	12.5	16.2
1999–2019	13.5	16.1	18.9	19.6	19.2	18.5	15.7	17.3
	**Solar radiation (W m**^−^**^2^)**							Total
2017	195	263	289	299	247	201	159	1653
2019	246	270	342	330	288	251	203	1930
1999–2019	225	271	306	309	273	227	161	1772
	**Precipitation (mm)**							Total
2017	85.3	0.0	7.4	0.0	0.0	1.0	4.6	98.3
2019	12.5	88.9	0.0	0.2	0.0	1.5	0.2	103.3
1999–2019	44.3	26.2	5.3	0.2	0.0	2.1	37.9	116.1
	**Minimum daily temperature (°C)**							Mean
2017	6.8	8.2	10.7	10.8	12.2	11.3	5.7	9.4
2019	8.8	8.4	11.2	11.1	12.3	9.7	4.9	9.5
1999–2019	6.5	8.2	9.9	11.0	10.9	9.4	7.5	9.1
	**Maximum daily temperature (°C)**							Mean
2017	21.5	26.3	29.3	31.3	30.5	30.4	27.9	28.2
2019	23.3	22.4	29.2	29.9	31.2	29.4	26.6	27.4
1999–2019	21.3	24.5	28.2	29.6	29.6	29.3	25.6	26.9
	**Days with temperature over 30 °C (no)**							Total
2017	0	1	10	13	16	11	13	64
2019	0	3	0	11	12	18	13	57

Weather data were obtained from the CIMIS weather station #77 (Oakville, CA) located at the research site.

### Canopy Architecture, Yield Components, and Labor Operations Costs of Experiment 3

Leaf area index (LAI) was measured on 21 June to characterize grapevine canopy growth and converted into leaf area on by a smartphone based program, VitiCanopy, coupled with an iOS system (Apple Inc., Cupertino, CA, USA) (De Bei et al., 2016). The gap fraction threshold was set to 0.75, extinction coefficient was set to 0.7, and sub-divisions were 25. A “selfie-stick” was used for an easy access to place the device about 75 cm underneath the canopy. The device was positioned with the maximum length of the screen being perpendicular to the cordon, and the cordon being in the middle of the screen according to previous work (De Bei et al., 2016; Yu and Kurtural, 2020). In each experimental unit, three images were taken to capture half canopy of each vine, and analyzed by the software. The relationship between leaf dry mass and area was determined on a subsample of leaves of different sizes using a leaf area meter (Li-Cor 3300, Lincoln, NE USA). Total leaf area was calculated by defoliating one grapevine per treatment replicate after harvest and using the regressive relationship between leaf dry mass and leaf area. At harvest, clusters were manually removed, counted, and weighed on a top-loading balance. Leaf area to fruit ratio was calculated by dividing leaf area with crop weight. Dormant pruning weight was collected during the dormant season (16 December); and crop load was calculated as the ratio between yield per vine (kg) and the pruning mass (kg) of each vine. Labor operations costs and gross income per hectare were calculated based on yield and net returns per hectare (California Department of Food and Agriculture, 2020; Kurtural et al., 2020) and methods presented elsewhere (Kurtural et al., 2019). Anthocyanin productivity (unit cost to produce anthocyanin) was calculated as reported by Cook et al. (2015).

### Berry Mass and Chemistry

At each sampling point and experiment, 55 berries were randomly collected from the middle of each treatment-replicate and kept on ice until they were measured. Berries were weighed, and mean berry mass was determined as the average mass of the counted berries. These berries were used to determine the total soluble solids (TSS), the pH, and the titratable acidity (TA). TSS was measured as °Brix, with a digital refractometer (Atago PR-32 Palette digital refractometer, ATAGO USA, Bellevue, WA, United States). The juice pH and TA was determined with an autotitrator (862 Compact Titrosampler, Herisau, Switzerland) using sodium hydroxide to titrate to an end point of pH 8.3, and it was expressed as g•L^−1^ of tartaric acid.

### Berry Flavonoid Content and Composition

For each sampling point in each experiment, 20 berries were collected, gently peeled, and berry skins were freeze-dried (Cold Trap 7385020, Labconco, Kansas City, MO, United States). Dried tissues were ground with a tissue lyser (MM400, Retsch, Germany). Fifty mg of the resultant powder was extracted in methanol: water: 7 M hydrochloric acid (70:29:1, V/V/V) to simultaneously determine flavonol and anthocyanin concentration and profile as previously described Martínez-Lüscher et al. (2019). Briefly, extracts were filtered (0.45 µm, Thermo Fisher Scientific, San Jose, CA, United States) and analyzed using an Agilent 1260 series reversed-phase high performance liquid chromatography (HPLC) system (Agilent 1260, Santa Clara, CA, United States) coupled to a diode array detector. Separation was performed on a reversed-phase C18 column LiChrospher^®^ 100, 250 mm × 4 mm with a 5-µm particle size and a 4-mm guard column of the same material at 25°C with elution at 0.5 ml per minute. The mobile phase was designed to avoid co-elution of anthocyanins and flavonols (Martínez-Lüscher et al., 2019) consisted in a constant 5% of acetic acid and the following gradient (v/v) of acetonitrile in water: 0 min 8%, at 25 min 12.2%, at 35 min 16.9, at 70 min 35.7%, 65% between 70 and 75 min, and 8% between 80 and 90 min. The identification of flavonoid compounds was conducted by determining the peak area of the absorbance at 280, 365, and 520 nm for flavan-3-ols, flavonols and anthocyanins, respectively. Identification of individual flavan-3-ols, anthocyanins, and flavonols were made by comparison of the commercial standard retention times found in the literature. Commercial standards of epicatechin, malvidin-3-O-glucoside, and quercetin-3-O-glucoside (Sigma-Aldrich, St. Louis, MO) were used for the quantification of flavan-3-ols, anthocyanins, and flavonols, respectively. The determination of proanthocyanidins was performed using an Agilent HPLC-DAD (1100 series, Agilent, Santa Clara, CA) after an acid catalysis in the presence of excess phloroglucinol (Kennedy and Jones, 2001), with minor modifications described in Martínez-Lüscher et al. (2017).

### Quantification of 3-Isobutyl-2-Methoxypyrazine With GC-MS

The 3-isobutyl-2-methoxypyrazine (IBMP) was quantified by a stable isotope dilution assay (SIDA) using headspace solid phase microextraction coupled to a gas chromatograph and a mass spectrometer (HS-SPME-GC-MS) as described Chapman et al. (2004) and Koch et al. (2010) with some modifications. Briefly, 20 berries per treatment-replicate from Experiment 3 were randomly collected from the clusters of three vines in the middle of each treatment-replicate on both side of the canopy, by cutting the pedicel with a pair of scissors and frozen at −80°C until analysis. Pedicels were removed by hand and berries were placed in 50 ml conical tubes. 10 ml of pure water and 100 μl of deuterated ([^2^H_3_]) IBMP isotope (5 pL•L^−1^) were added into the tube. Then, samples were ground with a tissue homogenizer Power Gen 1800D (Fisher Scientific, PA-USA) and centrifuged at 3000 rpm for 10 min. 10 ml of the supernatant was pipetted into 20 ml SPME vials containing 3 g of sodium chloride.

Samples were analyzed with an Agilent 6890N gas chromatograph equipped with a split/splitless injector coupled to a 5973 mass selective detector (MSD) (Agilent Technologies, Santa Clara, CA, USA). A Gerstel MPS2 autosampler (Gerstel Inc., Columbia, MD) and a HP 5MS capillary column (30 mm × 0.25 mm and 0.25 film thickness) were used for head space (HS) sampling. Then, HS samples were exposed to a 23-gauge, 2 cm divinylbencene/carboxen/polymethylsiloxane (DVB/CARB/PDMS) SPME fiber for 30 min at 40°C with continuous agitation for extraction. SPME fiber was desorbed at 260°C in splitless for 5 min into the GC-MS inlet with a 0.7 mm straight glass liner. Inlet flow was set to 50 ml•min^−1^ for another 5 min. Oven temperature was held at 40°C for 5 min, then ramped 2.5°C•min^−1^ up to 80°C, 5°C•min^−1^ up to 110°C 110°C, 25°C•min^−1^ up to 230°C and finally kept steady at 230°C for 5 min. The MSD interface was kept at 280°C and the carrier gas was Helium at a constant pressure of 4.77 psi with an initial flow of 0.8 ml•min^−1^. Selected ion monitoring was used at mass of m/z=124 for IBMP and m/z=127 for [^2^H_3_]IBMP.

### Statistical Analysis

Statistical analyses were carried out using the R-Studio version 3.6.1 (RStudio: Integrated Development for R., Boston, MA, USA) for Windows. All data were subjected to Shapiro-Wilk's normality test (Royston, 1995). Correlations between variables were calculated with the Pearson's test by using the same software. Segmented regression analysis was used to determine the point of inflection the in the relationship between increasing exposure (percent kaempferol as described in Martínez-Lüscher et al. (2019)) and the berry skin anthocyanin and flavonol content with “segmented” 0.5-0.3 R package (Muggeo, 2008). Data were normally distributed and, subsequently, were submitted to an analysis of variance (ANOVA) to assess the statistical differences between the treatments applied in each experiment performed. Means ± standard errors (SE) were calculated, and when the F value was significant (P≤ 0.05), a Duncan's new multiple range *post hoc* test was executed using “agricolae” 1.2-8 R package (de Mendiburu, 2016). When data were not normally distributed, a Kruskal-Wallis test was conducted. Percentage data were transformed according to the suggestion of the most likelihood test, into arcsine root square before ANOVA or Kruskal-Wallis tests.

## Results

### Effect of Different Solar Exposure on Berry Mass, Must Composition and Berry Skin Flavonoids

The growing season of 2017 was warmer and drier compared to the reference data for the same period within the last 20 years (**Table 1**). Thereby, average daily temperature was 4°C higher and rainfall was 18 mm less. Grape berry mass differed significantly depending on the degree of exposure (**Table 2**). Overexposed berries (Exp+ Deg+ and Exp+ Deg++) were the smallest due to overexposure resulting in dehydration thereby reducing berry mass. Neither total soluble solids nor titratable acidity changed regardless of the degree of exposure to which berries were subjected. However, the juice pH of the Exp+ Deg+ and Exp+ Deg++ berry must was greater (p=0.022) compared to Exp− and Exp+ Deg− berries.

**Table 2:** Effect of different degree of exposure on the berry mass and composition, and berry skin flavonoid profile of Cabernet sauvignon grapevine berry at harvest: i) Exp− (non-exposed berries collected from clusters in canopy interior), ii) Exp+ Deg− (exposed but not degraded, collected from Northeast exposed clusters), iii) Exp+ Deg+ (exposed and degraded, collected from Southwest exposed clusters), and iv) Exp+ Deg++ (exposed and very degraded grapes with signs of damage collected from Southwest exposed clusters) and collected in Oakville, CA in 2017.

	Degree of exposure				ANOVA
	Exp−	Exp+ Deg−	Exp+ Deg+	Exp+ Deg++	*P* value
Berry mass (g)	1.14 ± 0.04 a	1.18 ± 0.03 a	1.00 ± 0.03 b	0.86 ± 0.03 c	<0.0001
pH	3.40 ± 0.01 b	3.42 ± 0.01 b	3.55 ± 0.03 a	3.51 ± 0.06 ab	0.022
Titratable acidity (g•L^−1^)	7.98 ± 0.11	7.93 ± 0.14	7.40 ± 0.33	7.70 ± 0.51	ns
TSS (°Brix)	24.35 ± 0.4	23.63 ± 0.21	24.98 ± 0.76	25.03 ± 0.71	ns
Total anthocyanins (mg•berry^−1^)	2.23 ± 0.04 a	2.11 ± 0.11 a	0.63 ± 0.09 b	0.27 ± 0.02 c	<0.0001
Ratio 3'4'5'/3'4'	10.91 ± 0.38 a	9.30 ± 0.38 b	9.51 ± 0.29 b	7.74 ± 0.18 c	<0.0001
Total flavonols (mg•berry^−1^)	0.106 ± 0.006 b	0.196 ± 0.008 a	0.081 ± 0.008 c	0.054 ± 0.003 d	<0.0001
% Kaempferol	5.80 ± 0.47 c	9.08 ± 0.59 b	11.53 ± 0.55 a	13.23 ± 0.56 a	<0.0001
% Myricetin	35.71 ± 0.55 a	28.98 ± 0.92 b	20.97 ± 1.74 c	12.95 ± 0.86 d	<0.0001
% Quercetin	42.33 ± 0.76 d	49.24 ± 0.73 c	53.37 ± 1.59 b	61.56 ± 0.91 a	<0.0001
Total proanthocyanidins (mg•berry^−1^)	4.58 ± 0.1 a	4.98 ± 0.27 a	2.48 ± 0.18 b	1.94 ± 0.23 b	<0.0001
Total flavan-3-ols (mg•berry^−1^)	0.017 ± 0.001 a	0.016 ± 0.001 A	0.011 ± 0.000 b	0.009 ± 0.001 b	<0.0001

Values represent means separated by Duncan's new multiple range test (at P = 0.05). Within columns, means followed by different letters are significantly different as affected by the degree of exposure. ns, not significant (P > 0.05).

Berry skin flavonoid content and composition were also affected by the degree of exposure (**Table 2**). The berry anthocyanin content of Exp− was similar to Exp+ Deg−. However, overexposed berries resulted in berry anthocyanin content that was 70% and 90% lower when compared to the Exp− berries. Grape berry exposure to solar radiation not only affected the anthocyanin content but also modified the ratio between the tri- and di-substituted anthocyanins leading to a less stable profile in all treatments with exposed berries. Likewise, berry skin flavonol content and composition were strongly affected by the degree of exposure to solar radiation. Therefore, in Exp+ Deg− flavonol content was two-fold greater than Exp−, albeit they abruptly decreased in overexposed grapes (Exp+ Deg+ and Exp+ Deg++) where flavonol content was 25% and 50% lower when compared to Exp− berries. Furthermore, in overexposed berries the proportion of kaempferol and quercetin significantly increased while the proportion of myricetin decreased.

Regarding proanthocyanidins in berries, mild exposure did not affect their content in Exp+ Deg− compared to Exp− berries. However, greater solar exposure (Exp+ Deg+ and Exp+ Deg++) decreased proanthocyanidin content in berries but to a lesser extent compared to Exp−(45% and 60%, respectively). Finally, the content of flavan-3-ols was severely reduced in Exp+ Deg++ berries (47% lower than the flavan-3-ol content in Exp− berries).

### Assessing the Canopy Porosity Threshold for Optimum Berry Flavonoid Content

**Figure 2:**
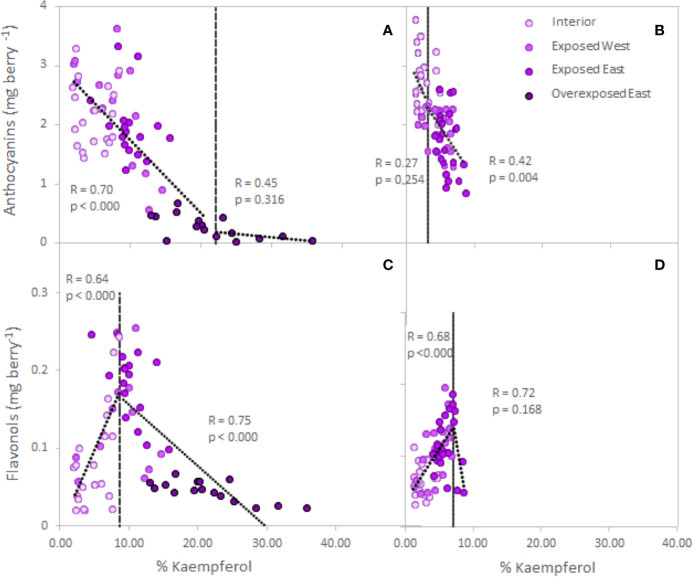
Relationship between grape skin anthocyanin **(A, B)** and flavonol **(C, D)** content (mg per berry) and increasing exposure (% of kaempferol, Martínez-Lüscher et al., 2019) in Cabernet Sauvignon **(A, C)** and Petit Verdot **(B, D)** single berries collected from the cluster interior (Exp−), exposed West (Exp+ Deg−), exposed East (Exp+ Deg+) and overexposed East (Exp+ Deg++). Grey dashed lines are the breaking points determined through segmented regression.

The analyses performed on single berries from two varieties confirmed the obtained response in anthocyanins and flavonols in Cabernet Sauvignon (**Figure 2**, **Table S1**). Thus, exposure affected the accumulation/degradation of these flavonoids. Exposed berries from the East side of the canopy decreased 8% and 36% of the anthocyanin content in Cabernet Sauvignon and Petit Verdot, respectively. Thus, Petit Verdot seemed to be more sensitive to higher level of solar exposure and degraded anthocyanins. Overexposed berries of Cabernet Sauvignon resulted in an 87% decrease of the berry skin anthocyanins when compared to the interior berries (**Table S1**). Berry skin anthocyanins and increasing exposure showed a significant trend below the 22% of kaempferol (**Figure 2A**). Conversely, analysis of the segmented regression on Petit Verdot berries did not show a clear trend below the 3.2% of Kaempferol and after the point of inflection, anthocyanins started to degrade (**Figure 2B**). Regarding flavonol content, no differences were observed between cultivars (cultivar, *p* = 0.978, **Table S1**). Conversely, when exposure increased to *ca*. 60% the content of flavonols in exposed berries of both canopy sides and in both cultivars; the overexposed berries had the lowest flavonol content (**Table S1**). Thus, our data revealed a strong positive relationship between the berry skin flavonols and the percentage of kaempferol until 8.6% of kaempferol proportion for Cabernet Sauvignon (R= 0.64, *p*< 0.0001) and 7.2% Petit Verdot (R= 0.68, *p*< 0.0001) (**Figures 2C, D**). However, beyond these thresholds, flavonols started to degrade, and there was an indirect relationship between the flavonol content and the percentage of kaempferol for both cultivars, this relationship being significant only for Cabernet Sauvignon (**Figures 2C, D**).

### Different Solar Exposure Driven by Canopy Management Affects Grapevine Performance and Berry Quality

The weather conditions during the execution of this experiment were highlighted by greater maximum daily temperatures when compared to the reference period (1999–2019). This was more prominent during the driest months (**Table 1**). Moreover, global solar radiation received at the experimental site was to *ca*. 200 W m^−2^ greater than the total solar radiation recorded within the reference period (**Table 1**).

The LR and ST decreased leaf area index (LAI) and increased canopy porosity. The combinatory effect of LR and LT treatments caused a 58% reduction of LAI and a 45% increase of canopy porosity (**Table 3**). However, neither leaf area nor pruning mass showed significant differences between treatments. On the other hand, yield components were mostly affected by the shoot thinning treatments (**Table 3**). Thus, shoot thinned vines showed lower number of clusters, yield, and Ravaz Index (RI), and increased leaf area to fruit ratio per vine as expected. The extent of yield reductions was 55% and 47% for ST and LRST vines, respectively (**Tables 3** and **4**).

**Table 3:** Effect of the canopy management practices untreated control (UNT), leaf removal (LR), shoot thinning (ST), and a combination of both (LRST) on the canopy architecture, and yield components of Cabernet sauvignon grown in Oakville, CA in 2019.

	Cultural practice				ANOVA
	UNT	LR	ST	LRST	*p* value
*Canopy architecture*					
LAI	1.68 ± 0.078 a	1.32 ± 0.152 b	1.18 ± 0.076 b	0.98 ± 0.113 c	<0.0001
Percent canopy porosity	21.0 ± 1.4 c	24.6 ± 1.7 b	26.8 ± 1.7 b	30.4 ± 1.4 a	<0.0001
Leaf area (m^2^∙vine^−1^)	5.70 ± 0.93	4.82 ± 0.49	4.76 ± 0.68	4.13 ± 0.81	ns
Pruning mass (kg∙vine^−1^)	1.38 ± 0.12	1.26 ± 0.14	1.08 ± 0.10	1.00 ± 0.12	ns
*Yield components*					
Clusters per vine	107 ± 10 a	95 ± 19 a	48 ± 2 b	44 ± 1 b	<0.0001
Cluster mass (g)	87.37 ± 3.91	86.83 ± 9.21	107.33 ± 16.08	99.56 ± 13.47	ns
Yield (kg∙vine^−1^)	9.36 ± 0.61 a	8.63 ± 1.55 a	5.12 ± 0.88 b	4.43 ± 0.58 b	<0.0001
Leaf area to fruit ratio (m^2^∙kg^−1^)	0.60 ± 0.03 b	0.57 ± 0.04 b	0.93 ± 0.02 a	0.93 ± 0.07 a	<0.0001
Ravaz Index (RI) (kg∙kg^−1^)	6.87 ± 0.77 a	6.90 ± 0.68 a	4.76 ± 0.18 b	4.52 ± 0.67 b	<0.0001

Values represent means separated by Duncan's new multiple range test (at P = 0.05). Within columns, means followed by different letters are significantly different as affected by the canopy management practices of leaf removal and shoot thinning and their interactions. ns, not significant (P > 0.05).

**Table 4:** Cost estimates on labor operations costs of canopy management practices and cost to produce one unit of anthocyanin and IBMP of Cabernet Sauvignon grapevine subjected to untreated control (UNT), leaf removal (LR), shoot thinning (ST), and a combination of both (LRST) in Oakville, CA in 2019 (Kultural et al., 2020).

		Cultural practice				ANOVA
		UNT	LR	ST	LRST	*P* value
*Cultural practices labor operation cost ($/Ha)*						
Dormant pruning		1,336.27	1,336.27	1,336.27	1,336.27	–
Shoot thinning		0	0	738.53	738.53	–
Leaf removal		0	2,067.39	0	2,067.39	–
Total		1,336.27	3,403.66	2,074.8	4,142.19	–
Yield (Mg/Ha)		19.5 ± 0.64 a	17.97 ± 1.62 a	10.66 ± 0.92 b	9.23 ± 0.61 b	<0.0001
Gross income ($/Ha)		$170,702	$157,308	$93,317	$80,798	–
Anthocyanin productivity ($/kg)		29.69 ± 1.78 c	92.09 ± 7.57 b	92.10 ± 7.57 b	223.42 ± 0.42 a	<0.0001
IBMP productivity ($/µg)		15.80 ± 0.45 b	42.37 ± 5.76 b	53.41 ± 12.55 b	180.74 ± 26.69 a	<0.0001

Values represent means separated by Duncan's new multiple range test (at p = 0.05). Within columns, means followed by different letters are significantly different as affected by the canopy management practices of leaf removal and shoot thinning and their interactions.

**Figure 3:**
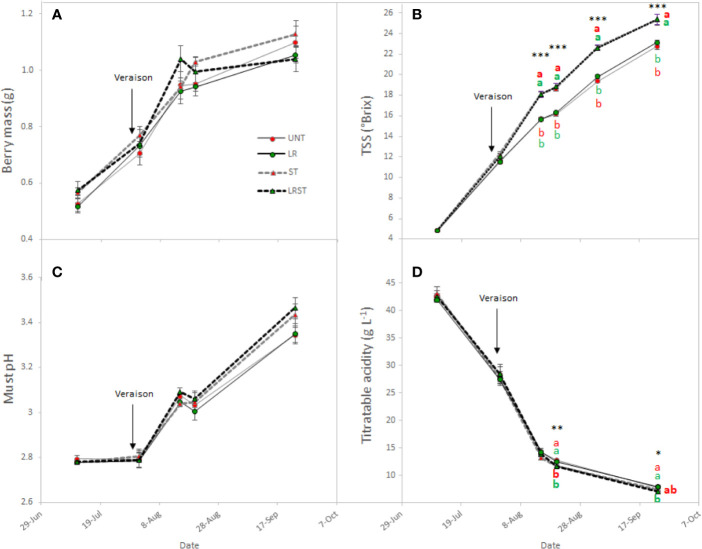
Effect of canopy management practices (UNT, untreated; LR, leaf removal; ST, shoot thinning and LRST, LR and ST combined) on berry mass **(A)** Brix **(B)**, must pH **(C)**, and titratable acidity **(D)** the growing season. Values represent means ± SE (n = 4). At each time point, different letters indicate significant differences (*p* < 0.05) between canopy management practices according to the one-way ANOVA followed by Duncan's new multiple range test. *, **, and *** indicate significance at 5%, 1%, and 0.1% probability levels, respectively.

Berry mass was not significantly affected by canopy management practices during the berry ripening although vines subjected to LRST tended to result in smaller berries (**Figure 3A**). The most influential effects observed on berry chemistry were due to shoot thinning treatments (**Figure 3**). Therefore, shoot thinned vines had greater total soluble solids and lower titratable acidity from mid-ripening to harvest. However, no significant effect was observed on the must pH (**Figures 3B–D**).

**Figure 4:**
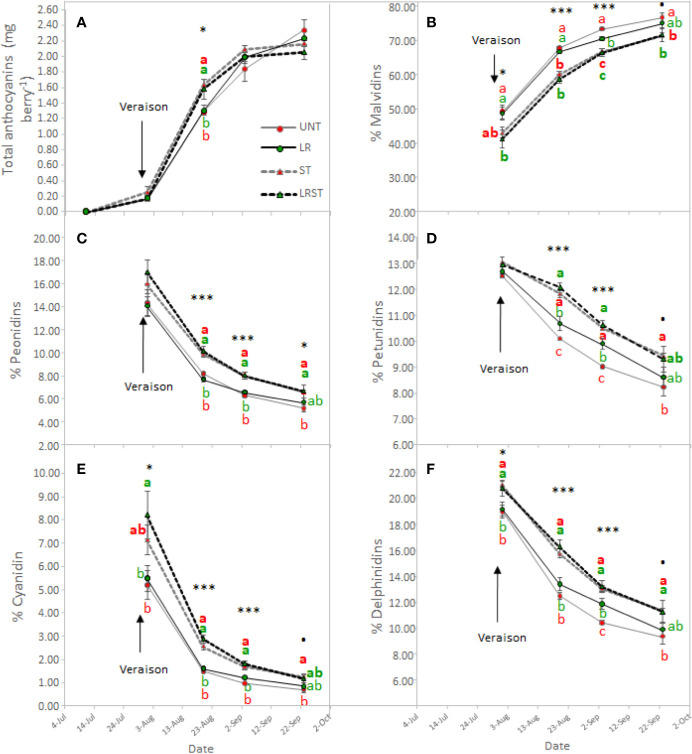
Effect of canopy management practices (UNT, Untreated; LR, Leaf removal; ST, Shoot thinning; LRST, LR and ST combined) on berry skin anthocyanin content **(A)**, percent Malvidin **(B)**, percent Peonidin **(C)**, percent Petunidin **(D)**, percent Cyanidin **(E)** and percent Delphinidin **(F)** during the growing season. Values represent means ± SE (n = 4). At each time point, different letters indicate significant differences (*p* < 0.05) between canopy management practices according to the one-way ANOVA followed by Duncan's new multiple range test. **·**,*, and *** indicate significance at 10%, 5%, and 0.1% probability levels, respectively.

**Figure 5:**
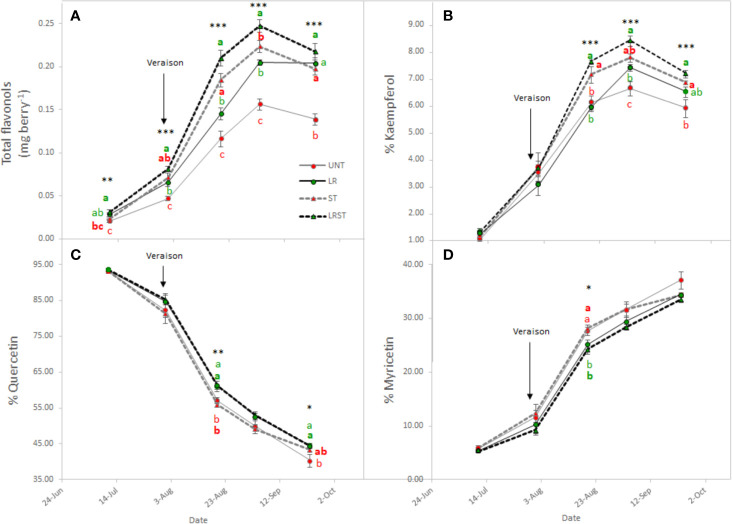
Effect of canopy management practices (UNT: Untreated, LR: Leaf removal, ST: Shoot thinning and LRST: LR and ST combined) on berry skin flavonol content **(A)**, percent Kaempferol **(B)**, percent Quercetin **(C)**, and percent Myricetin **(D)** during the growing season. Values represent means ± SE (n = 4). At each time point, different letters indicate significant differences (*p* < 0.05) between canopy management practices according to the one-way ANOVA followed by Duncan's new multiple range test. *, **, and *** indicate significance at 5%, 1%, and 0.1% probability levels, respectively.

Shoot thinned grapevines had higher anthocyanin content at veraison (**Figure 4A**). However, we did not measure any changes to anthocyanin content at harvest as affected by the canopy management practices applied. Although anthocyanin content was not affected, anthocyanin composition was modified by treatments from mid-ripening to harvest (**Figure 4B–F**). Berry skins of ST and LRST grapevines showed a lower 3'4'5'/3'4' ratio leading to increased proportion of cyanidins and peonidins (**Figures 4C, E**) in detriment of malvidins which was the most abundant anthocyanin found in berry skins (**Figure 4B**). During the monitored period, different canopy management practices modified berry flavonol content (**Figure 5A**). The berries from LRST grapevines showed the greatest berry skin flavonol content, while, at harvest, the flavonol content of LR, ST, and LRST was similar and greater when compared to the UNT content. Not only canopy management practices modified flavonol content but they also affected their composition. The LRST treatment had a higher proportion of kaempferol and quercetin from mid-ripening to harvest (**Figures 5B, C**) and lower of proportion of myricetin after veraison (**Figure 5D**).

**Figure 6:**
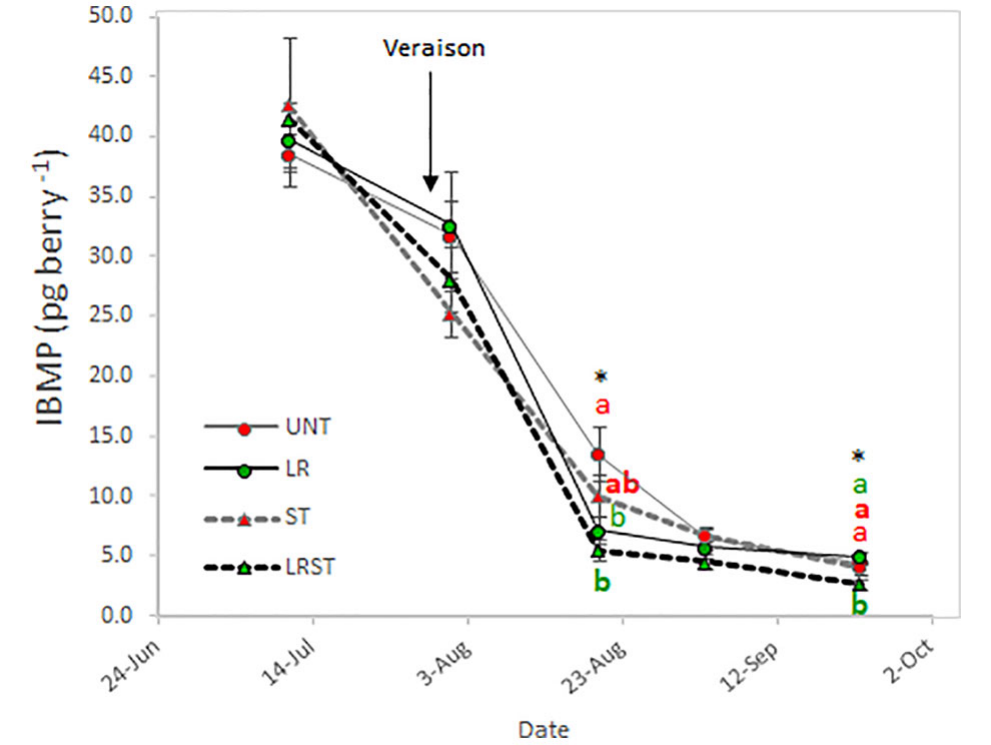
Effect of canopy management practices (UNT: Untreated, LR: Leaf removal, ST: Shoot thinning and LRST: LR and ST combined) on berry IBMP content during the growing season. Values represent means ± SE (n = 4). At each time point, different letters indicate significant differences (*p* < 0.05) between canopy management practices according to the one-way ANOVA followed by Duncan's new multiple range test. * indicate significance at 5% probability level.

As expected, berry IBMP content decreased throughout ripening with all the canopy management practices tested in this study (**Figure 6**). However, we found the significant differences among treatments after veraison and at harvest. The LRST treatment resulted in the lowest IBMP content from mid-ripening to harvest.

**Figure 7:**
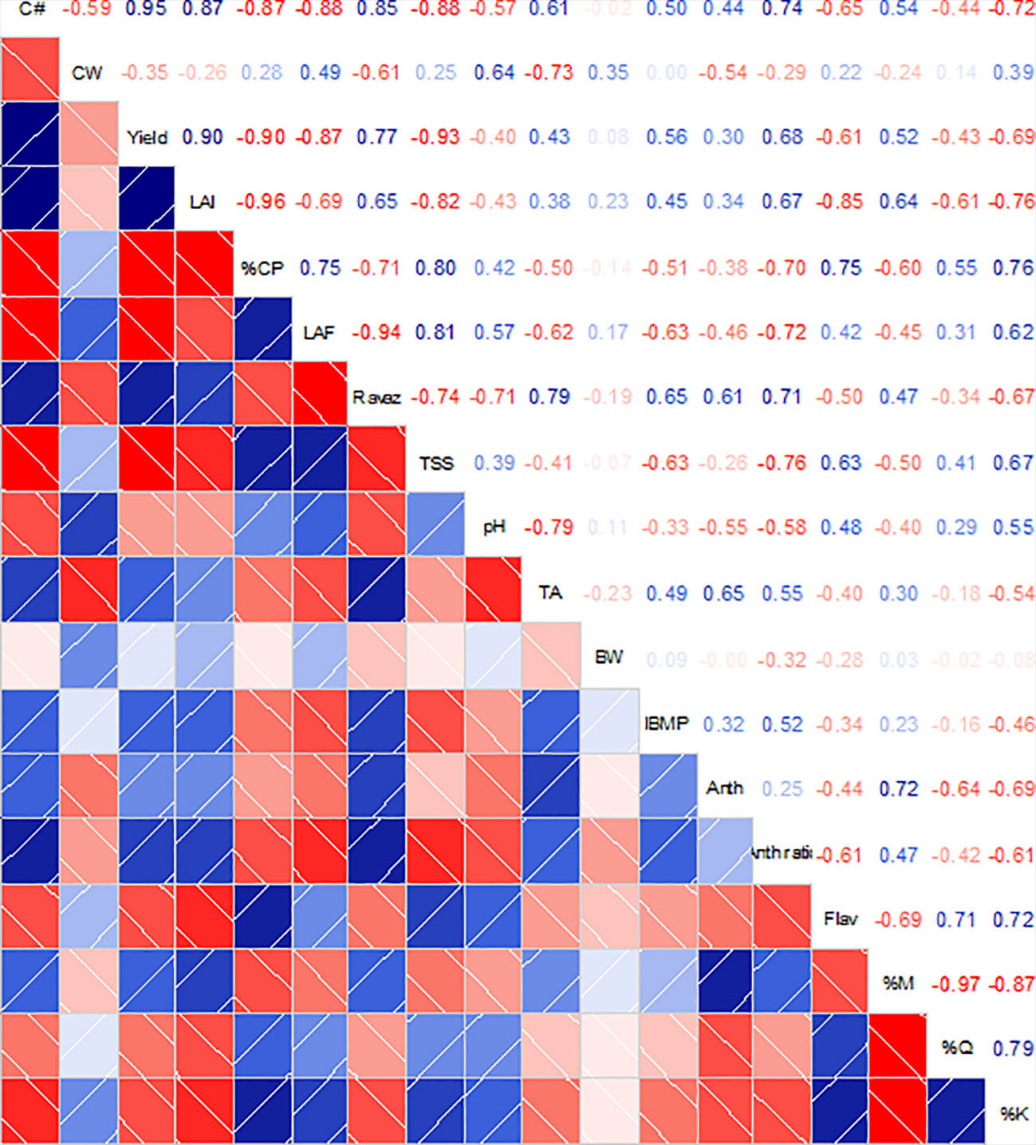
Correlation matrices among grapevine canopy architecture, yield components, berry mass and must compositions and flavonoid content and profile from Cabernet Sauvignon grapevines subjected to different canopy management practices (UNT, untreated; LR, Leaf removal; ST, Shoot thinning; LRST, LR and ST combined) at harvest. Upper panel shows the R values for the Pearson's correlation analysis. Intensity of blue or red colors in the upper and lower panels represents the significance of the relationship between variables. White lines in lower panel represent the regression curves for each pair of variables. C#, cluster number; CW, cluster mass; LAI, Leaf Area Index; %CP, % canopy porosity; LAF, leaf area to fruit ratio; RI, Ravaz Index; TSS, total soluble solids; TA, titratable acidity; BW, berry mass; Anth, total anthocyanins; Flav, total flavonols; %M, % myricetin; %Q, % quercetin; %K, % kaempferol; %Dp, % delphinidin; %Cy, % Cyanidin; %Pt, % Petunidin; %Pn, % Peonidin; %Mv, % Malvidin.

Correlation analysis between the monitored variables at harvest revealed a strong relationship between canopy architecture variables (LAI and canopy porosity) and berry flavonol content (**Figure 7**). Moreover, canopy porosity was strongly correlated to the kaempferol proportion in berry skins (r = 0.75, *p* = 0.001). On the other hand, a lower yield due to canopy management practices was related to decreased IBMP and increased flavonol content (r = 0.56, *p* = 0.025 and r = −0.61, p = 0.012, respectively). Finally, a strong relationship was found between TSS and TA with the leaf to fruit ratio (r = 0.81, *p* < 0.001 and r = −0.62, *p* = 0.011). Finally, a higher solar exposure estimated as the kaempferol proportion was strongly correlated with decreased anthocyanin berry contents (r = −0.69, *p* = 0.003) and yield (r = −0.69, *p* = 0.002).

Analysis of labor operations cost of canopy management practices indicated that the most expensive canopy management practices was the LRST (**Table 4**) where growers received a 53% lower income per hectare. Thereby, productivity data provided evidence that the cost of producing a kg of anthocyanin and removing a µg of IBMP was 10-fold greater in LRST compared to UNT per ha (**Table 4**).

## Discussion

### Effects of Canopy Management Practices on Canopy Architecture and Yield Components

Yield components were mainly affected by shoot thinning practices, decreasing the number of clusters and yield per vine leading to unbalanced vines (RI < 5) according to the previous studies (Sun et al., 2012; Jogaiah et al., 2013). Yield per meter of row is increased quasilinearly with the increase in shoot density per meter of row as indicated by previous studies (Terry and Kurtural, 2011; Geller and Kurtural, 2013). The lack of effect of LR on yield was corroborated by several studies (Pastore et al., 2013; Mijowska et al., 2016; Yu et al., 2016) when a late leaf removal was applied. Moreover, Yu et al. (2016) and Cook et al. (2015) reported that grapevines may produce more leaves than required, especially in warm climates, therefore, the increase in canopy gaps and the diminution of external leaf layers did not elicit decreases in yield as they were not severe enough reductions to the functional leaf area. The RI between 5 and 10 is considered optimum for vine balance (Bravdo et al., 1985; Terry and Kurtural, 2011). Therefore, RI and leaf area to fruit ratio data reported with the grapevines subjected to shoot thinning (ST and LRST) were under cropped that led to lower yields.

In our study, Cabernet Sauvignon vines were not able to modulate their vegetative biomass in response to canopy management practices applied. Previous studies showed that pruning mass values up to 1 kg/m of row were considered optimal under warm climate (Terry and Kurtural, 2011). In our experiment the pruning mass per meter of all treatments ranged from 0.5 (in LRST) to 0.7 (in UNT) kg/m without differences between treatments. Moreover, although the shoot counts were obviously different between treatments, we did not find differences in the pruning mass, that suggested lower lateral expansion and/or reduced shoot diameter with an increasing number of shoots as previously reported Brillante et al. (2018). Consequently, we found that the mass of each shoot ranged from 28 and 25 g in UNT and LR, respectively, to 45 and 42 g in ST and LRST, respectively, corroborating work by Brillante et al. (2018).

### Effects of the Cluster Microclimate on the Physico-Chemical Attributes of Berries

Martínez-Lüscher et al. (2017) reported negligible variation of berry mass of Cabernet Sauvignon due to higher solar exposure under irrigated viticulture. Similarly, berry masses remained unaffected by a higher solar exposure of the cluster due to canopy management practices unless they were directly exposed to sunlight where berries may suffer dehydration as previously reported by Mijowska et al. (2016). This has been attributed to the effect of the higher temperatures with subsequent increases in berry transpiration that affected cell division and elongation (reviewed by Uhlig, 1998).

Under our experimental conditions, shoot thinning treatments hastened berry ripening by enhancing the TSS to *ca*. 2.5°Brix and decreasing must titratable acidity by 0.6 g•L^−1^ at harvest. Thus, overexposure has been related with higher pH due to the elevated temperature that berries overcome and the subsequent organic acid degradation (Sweetman et al., 2014). Nevertheless, Wang et al. (2019) recently suggested that changes on the source-to-sink ratio induced by shoot thinning might have more influence on berry maturity than the change in the microclimate (higher light interception and canopy porosity) they reported.

### Effects of the Cluster Microclimate on the Berry Flavonoid Content and Profile and IBMP Content

Cultural practices have been related to increased anthocyanin content (Diago et al., 2012; Gatti et al., 2012; Bubola et al., 2017). However, in agreement with other studies (Sivilotti et al., 2016; Pastore et al., 2017), under our experimental conditions, berry anthocyanin content did not increase due to LR, ST or LRST. Similarly, anthocyanin content was not affected by mild-exposure in berries collected from the commercial vineyard either. Increasing exposure was detrimental for anthocyanin content as the overexposed berries were subjected to higher temperatures that may have impaired their accumulation (Martínez-Lüscher et al., 2017). The anthocyanin berry content at harvest is the result between synthesis and degradation rates. It was reported anthocyanin synthesis may be up-regulated by greater exposure (Matus et al., 2009). Therefore, ST and LRST increased the anthocyanin content at mid-ripening because of the increasing solar exposure (higher kaempferol proportion). Additionally, it was recently highlighted that some members of the dihydroflavonol reductase and UFGT genes required for anthocyanin biosynthesis were moderately up-regulated in LR treated berries leading to increases of anthocyanin content at mid-ripening (Sun et al., 2017). However, at harvest, no significant effect of canopy management practices on anthocyanin content was found, and this result is corroborated by Pastore et al. (2017) who reported no beneficial effect due to higher cluster exposure in warm climates. Although cultural practices may induce different cluster temperatures by increasing exposure, we did not find a clear relationship between exposure (% of kaempferol) and cluster temperature when kaempferol proportion are low (**Figure S2**) suggesting that results of this work were mainly explained by different exposures. Nevertheless, under elevated temperatures, a down-regulation of anthocyanin biosynthesis and enhanced rates of degradation have been reported (Movahed et al., 2016). Those authors suggested that high temperature induced anthocyanin degradation by enhancing the expression of VviPrx31 and consequently the peroxidase activity. Likewise, overexposed berries (Exp+ Deg+ and Exp+ Deg++) with kaempferol proportions greater than 10% were subjected to higher temperatures that dramatically decreased anthocyanin content.

Matus et al. (2009) reported that flavonol content increased by two-fold in exposed berries compared to non-exposed. Our results corroborated this finding partially, depending on the level and duration of exposure, canopy position of the berries, and orientation of the vineyard. Therefore, when flavonol proportion was below 10% of kaempferol, flavonol content increased; but would decrease after this inflection point due to degradation. Matus et al. (2009) further indicated that this increase in flavonol may be driven by the up-regulation of MYB12 and flavonols synthase 4 (FLS4) due to the greater exposure suggesting that FLS4 could be a target of MYB12 in grapevine. Accordingly, Sun et al. (2017) found that increased accumulation of flavonols in light exposure berries, were accompanied by the up-regulation of several genes of the FLS gene family suggesting that they may be functionally redundant in response to light signal.

During the experiment conducted in the 2019 growing season, the kaempferol proportion increased in LR and ST treatments, but largest increase was measured when ST and LR were applied concurrently. Likewise, the higher the degree of exposure degree a greater kaempferol accumulation was observed during the 2017 growing season. The increase in kaempferol in total proportion of flavonols was accompanied with a concomitant decrease of quercetin and myricetin proportions. These results are corroborated with our previous work performed on Merlot and Cabernet Sauvignon. (Martínez-Lüscher et al., 2019), and by others on Cabernet Sauvignon, Nero d'Avola, Raboso Piave, and Sangiovese in Italy (Pastore et al., 2017). We previously reported the proportion of kaempferol was a feasible tool for accounting the solar radiation received by berry due to the greater canopy porosity (Martínez-Lüscher et al., 2019) and this corresponded to the 1930 W·m^−2^ of global radiation accumulated at the research site in Experiment 3. On the other hand, the higher proportion of quercetin derivatives in detriment of myricetin derivatives found in LR vines has been related to downregulation of F3'5'H family genes (Sun et al., 2017).

Previous work on red grapevine berries, indicated that IBMP content decreased with greater solar exposure due to the canopy management practices during berry ripening (Ryona et al., 2008; Dunlevy et al., 2013). In our work, the lowest IBMP content was measured in LRST berries. Our results indicated a negative and linear relationship between leaf to fruit ratio and IBMP content. Conversely, the relationship between kaempferol proportion and IBMP was not significant. Therefore, our data suggested that the decrease of IBMP content was better explained by changes in the source-sink balance rather than differences in solar exposure. Likewise, Koch et al. (2012) provided evidence that solar exposure affected IBMP content to a greater extent when canopy porosity was enhanced before fruit set and not during berry ripening corroborating our results. The lower berry IBMP content was explained by a diminution of the accumulation rates rather than increased rates of degradation (Ryona et al., 2008) due to canopy management practices and restriction of applied water between fruit set and veraison (Brillante et al., 2018) in a warm climate.

### Labor Operations Costs of Canopy Management Practices

The total operating costs per hectare of a Cabernet Sauvignon vineyard in Napa County, CA U.S.A. is approximately US$ 40,382 (Kurtural et al., 2020). The labor operations costs of canopy management practices per hectare are 25% of the total costs. Our data indicated that although some berry traits were improved by the removal of shoots (2.5% increase in TSS) and leaves or the more common practice of doing them concurrently, their profitability is not ensured in warm climates. The unit cost to produce one unit of anthocyanin increased by about 10-fold with the additional canopy management practices. Therefore, the unfavorable leaf area to fruit ratios increased the cost of producing anthocyanins as previously reported by Cook et al. (2015) in Merlot grapevine grown in a warm climate. Likewise, the diminution of accumulation rates of IBMP were not as economically effective as once thought due to loss in yield and reduction in gross income per hectare for the grower. Finally, the breaking points determined through segmented regression analysis indicated that although increases in solar exposure (kaempferol proportion greater than 6.4% and 7% for anthocyanins and IBMP, respectively) led to significant IBMP content decreases (r = −0.95, *p* = 0.011), however, we were unable to elucidate this effect on anthocyanin content (r =−0.24, *p*=0.434, **Figure S3**).

## Conclusion

Since the effect of canopy management practices lead to higher solar exposure in hot climates that might be deleterious on grape quality, we aimed to elucidate the thresholds for maintaining anthocyanin content, while waiting for the target TSS required for fermentations and green aroma removal without compromising the yield. Although increasing canopy porosity through canopy management practices can be helpful for other purposes such as pest protection, this may not be the case of flavonoid compounds when a certain proportion of kaempferol is attained. Our data from these trials revealed different sensitivities to degradation within the flavonoid groups, flavonols being the only monitored compounds that were upregulated by solar radiation. Anthocyanin depletion was observed in all the trials with increasing solar radiation exposure (*i.e.* greater proportion of kaempferol). Under our experimental conditions, ST and LRST hastened fruit maturity; however, a clear improvement of the flavonoid compounds (*i.e.* greater anthocyanin content) was not observed at harvest. On the other hand, all the canopy management practices studied (LR, ST, and LRST) decreased IBMP from mid-ripening to harvest. Therefore, although some berry traits (i.e., increase of 2.5°Brix and lower IBMP content) were improved due to canopy management practices (ST and LRST), this came with costs of labor and yield and gross income reduction that decreased flavonoid productivity per hectare; and these all should be assessed together when taking the decision to apply these treatments in hot climates.

## Data Availability Statement

The raw data supporting the conclusions of this article will be made available by the authors, without undue reservation.

## Author Contributions

SK acquired the funding. NT, JM-L, EP, and SK conducted the experiments and analyzed the data. NT wrote the first version of the manuscript. All authors contributed to the article and approved the submitted version.

## Funding

The authors acknowledge the USDA-NIFA Specialty Crop Research Initiative award no. 2015-51181-24393, University of California Agriculture and Natural Resources Cooperative Extension Specialist Funds, Department of Viticulture and Enology Harold Olmo Research Trust, Rossi Endowment. The authors declare that this study received funding from Syngenta Crop Protection USA LLC and Allied Grape Growers. The funders were not involved in the study design, collection, analysis, and interpretation of data, the writing of this article or the decision to submit it for publication. The authors also acknowledge Constellation Brands United States for in-kind support and access to vineyards during the execution of the trial.

## Conflict of Interest

The authors declare that the research was conducted in the absence of any commercial or financial relationships that could be construed as a potential conflict of interest.

## Supplementary Material

Figure S1 Relationship between the % of Kaempferol and the % of Canopy porosity from berries collected from Cabernet Sauvignon **(A)** and Petit Verdot **(B)** varieties at harvest in Oakville in September 2017.

Figure S2 Relationship between the % of Kaempferol and cluster temperature at mid ripening in Cabernet Sauvignon subjected to different canopy management practices (UNT: Untreated, LR: Leaf removal, ST: Shoot thinning and LRST: LR and ST combined). Cluster temperature means separated by Duncan's new multiple range test (at P = 0.05). Within columns, means followed by different letters are significantly different as affected by the canopy management practices of leaf removal and shoot thinning and their interactions.

Figure S3 Relationship between grape skin anthocyanin (A) and IBMP (B) content (mg and pg per berry, respectively) and increasing exposure (% of kaempferol, Martínez-Lüscher et al., 2019) in Cabernet Sauvignon subjected to different canopy management practices (UNT: Untreated, LR: Leaf removal, ST: Shoot thinning and LRST: LR and ST combined). Black lines are the breaking points determined through segmented regression.

Table S1 Effect of the different degree of exposure on the skin flavonoid content of Cabernet Sauvignon and Petit Verdot berries collected from different orientations (Interior, Exposed from the West side of the canopy, Exposed from the East side and Overexposed from the East side of the canopy) in Oakville, CA in 2017. Values represent means separated by Kruskal-Wallis test (at *p* = 0.05). Within columns, means followed by different letters are significantly different as affected by the combination of degree of exposure and cultivar
