# Supplementary figures and images for: Optimal Ranges and Thresholds of Grape Berry Solar Radiation for Flavonoid Biosynthesis in Warm Climates
Source: Front Plant Sci. 2020 Jun 23;11:931. doi: 10.3389/fpls.2020.00931 (PMC7344324; doi:10.3389/fpls.2020.00931)

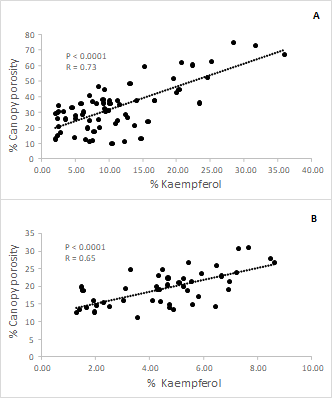

Supplement: Figure S1 — Relationship between the % of Kaempferol and the % of Canopy porosity from berries collected from Cabernet Sauvignon (A) and Petit Verdot (B) varieties at harvest in Oakville in September 2017. [file Image_1.tif]

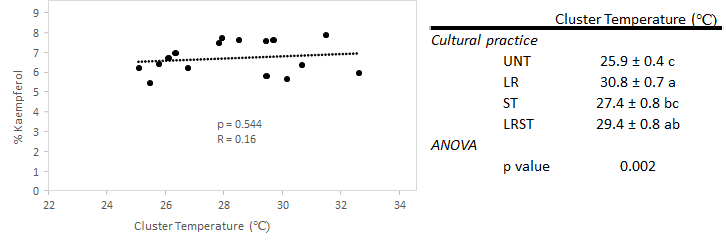

Supplement: Figure S2 — Relationship between the % of Kaempferol and cluster temperature at mid ripening in Cabernet Sauvignon subjected to different canopy management practices (UNT: Untreated, LR: Leaf removal, ST: Shoot thinning and LRST: LR and ST combined). Cluster temperature means separated by Duncan's new multiple range test (at P = 0.05). Within columns, means followed by different letters are significantly different as affected by the canopy management practices of leaf removal and shoot thinning and their interactions. [file Image_2.tif]

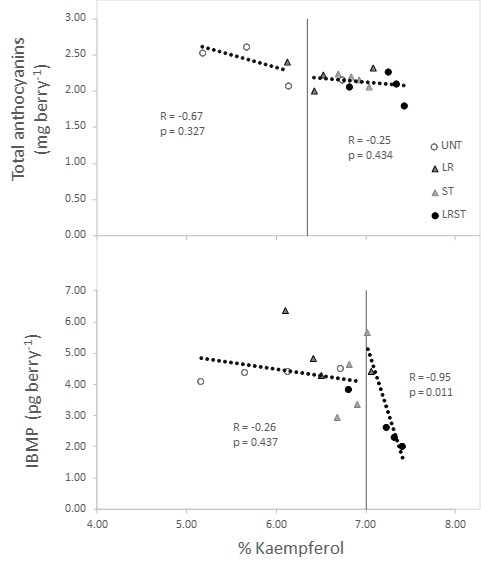

Supplement: Figure S3 — Relationship between grape skin anthocyanin (A) and IBMP (B) content (mg and pg per berry, respectively) and increasing exposure (% of kaempferol, Martínez-Lüscher et al., 2019) in Cabernet Sauvignon subjected to different canopy management practices (UNT: Untreated, LR: Leaf removal, ST: Shoot thinning and LRST: LR and ST combined). Black lines are the breaking points determined through segmented regression. [file Image_3.tif]
