# Supplementary material for: Optimal Ranges and Thresholds of Grape Berry Solar Radiation for Flavonoid Biosynthesis in Warm Climates
Source: Front Plant Sci. 2020 Jun 23;11:931. doi: 10.3389/fpls.2020.00931 (PMC7344324; doi:10.3389/fpls.2020.00931)
Supplement: Table S1 — Effect of the different degree of exposure on the skin flavonoid content of Cabernet Sauvignon and Petit Verdot berries collected from different orientations (Interior, Exposed from the West side of the canopy, Exposed from the East side and Overexposed from the East side of the canopy) in Oakville, CA in 2017. Values represent means separated by Kruskal-Wallis test (at p = 0.05). Within columns, means followed by different letters are significantly different as affected by the combination of degree of exposure and cultivar [file Table_1.doc]

**Table S1:** Effect of the different degree of exposure on the skin flavonoid content of Cabernet Sauvignon and Petit Verdot berries collected from different orientations (Interior, Exposed from the West side of the canopy, Exposed from the East side and Overexposed from the East side of the canopy) in Oakville, CA in 2017.

|  | Total anthocyanins (mg berry ^-1^) | Total flavonols (mg berry ^-1^) |
| --- | --- | --- |
| *Cabernet Sauvignon* |  |  |
| Interior | 2.16 ± 0.13 bc | 0.083 ± 0.014 cd |
| Exposed West | 2.27 ± 0.11 b | 0.130 ± 0.017 b |
| Exposed East | 1.98 ± 0.13 bcd | 0.183 ± 0.011 a |
| Overexposed East | 0.28 ± 0.05 e | 0.045 ± 0.003 e |
| *Petit Verdot* |  |  |
| Interior | 2.74 ± 0.10 a | 0.066 ± 0.005 de |
| Exposed West | 1.85 ± 0.09 cd | 0.109 ± 0.009 bc |
| Exposed East | 1.75 ± 0.12 d | 0.117 ± 0.009 b |
| *Kruskal-Wallis (p value)* |  |  |
| Cultivar | 0.019 | 0.978 |
| Orientation | < 0.0001 | < 0.0001 |
| Cultivar × Orientation | < 0.0001 | < 0.0001 |

Values represent means separated by Kruskal-Wallis test (at *p* = 0.05). Within columns, means followed by different letters are significantly different as affected by the combination of degree of exposure and cultivar
